# Supplementary material for: Flexible Mechanical Sensors Fabricated with Graphene Oxide-Coated Commercial Silk
Source: Nanomaterials (Basel). 2024 Jun 8;14(12):1000. doi: 10.3390/nano14121000 (PMC11206569; doi:10.3390/nano14121000)
Supplement: Supplementary file 1 [file nanomaterials-14-01000-s001.zip › nanomaterials-3044501-supplementary.docx]

**Supplementary Materials**

Flexible Mechanical Sensors Fabricated with Graphene Oxide-Coated Commercial Silk

Hyun-Seok Jang^1^, Ki Hoon Lee^1^, Byung Hoon Kim^1,2,3,^*

^1^Department of Physics, Incheon National University, Incheon, 22012, Republic of Korea

^2^Intelligent Sensor Convergence Research Center, Incheon National University, Incheon, 22012, Republic of Korea.

^3^ Institute of Basic Science, Incheon National University, Incheon, 22012, Republic of Korea.


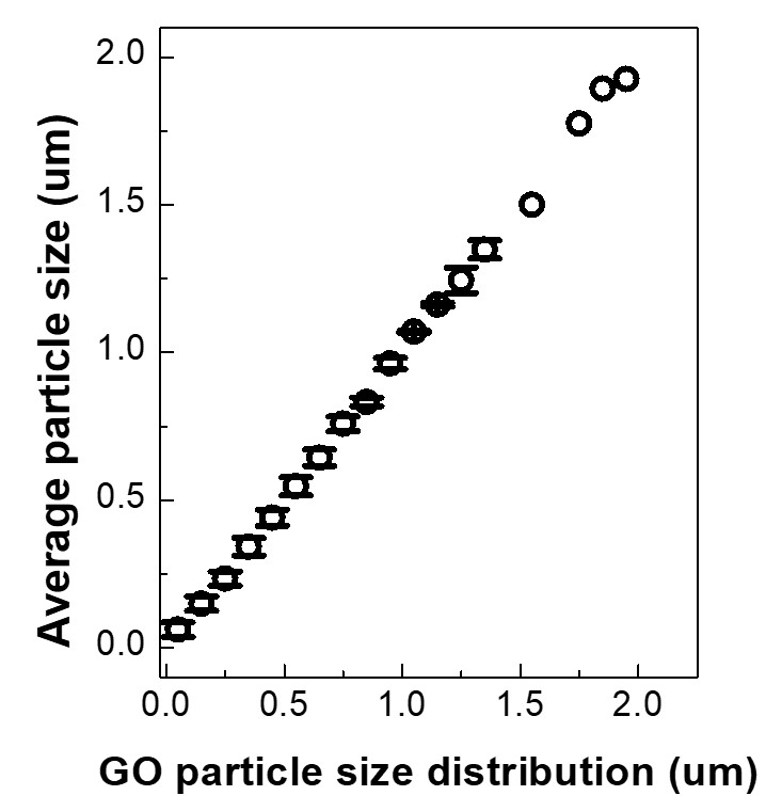


Figure S1. Average particle size and its standard deviation.


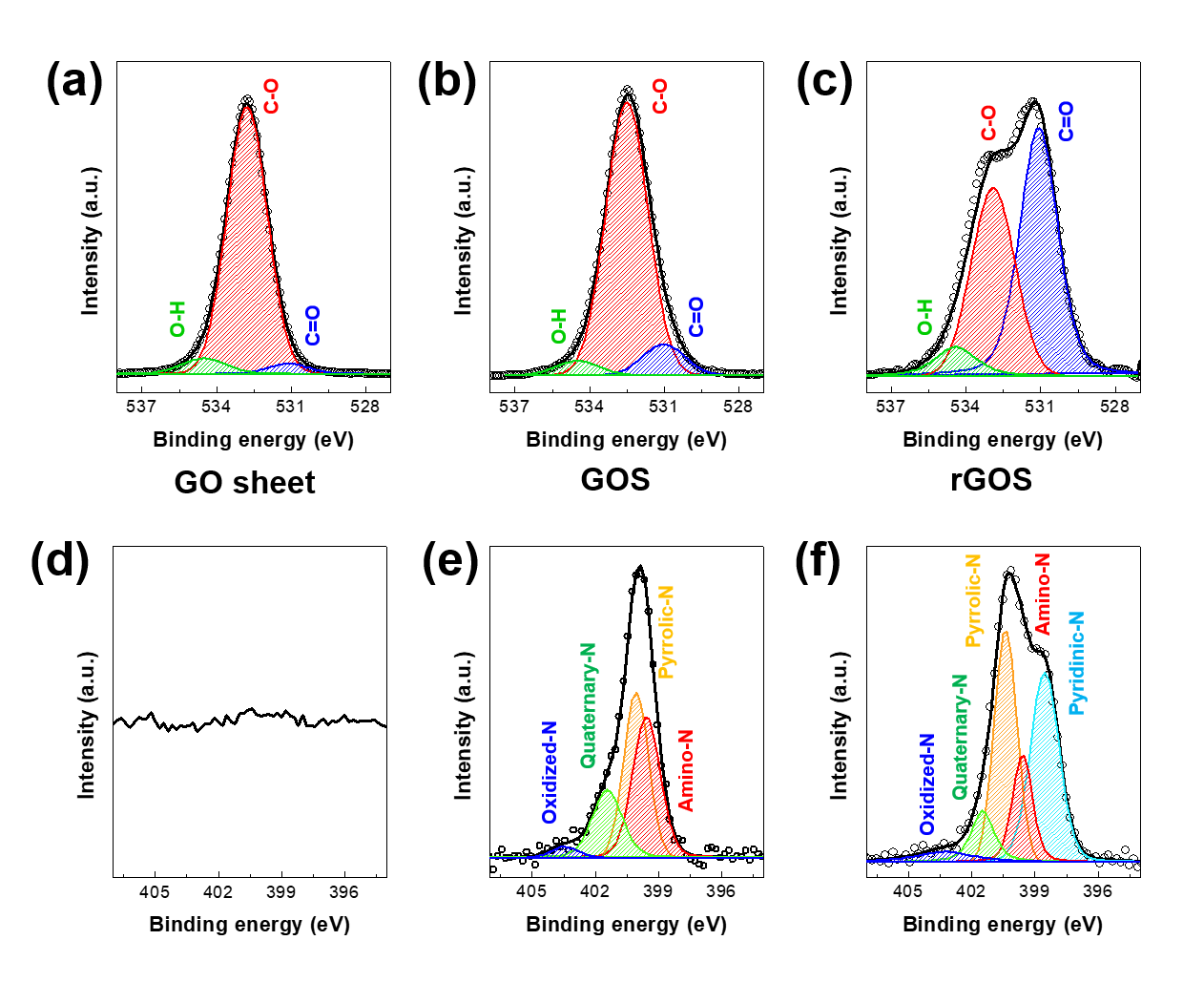


Figure S2. O1s XPS spectra of (a) GO, (b) GOS, and (c) rGOS. N1s XPS spectra of (d) GO, (e) GOS, and (f) rGOS.


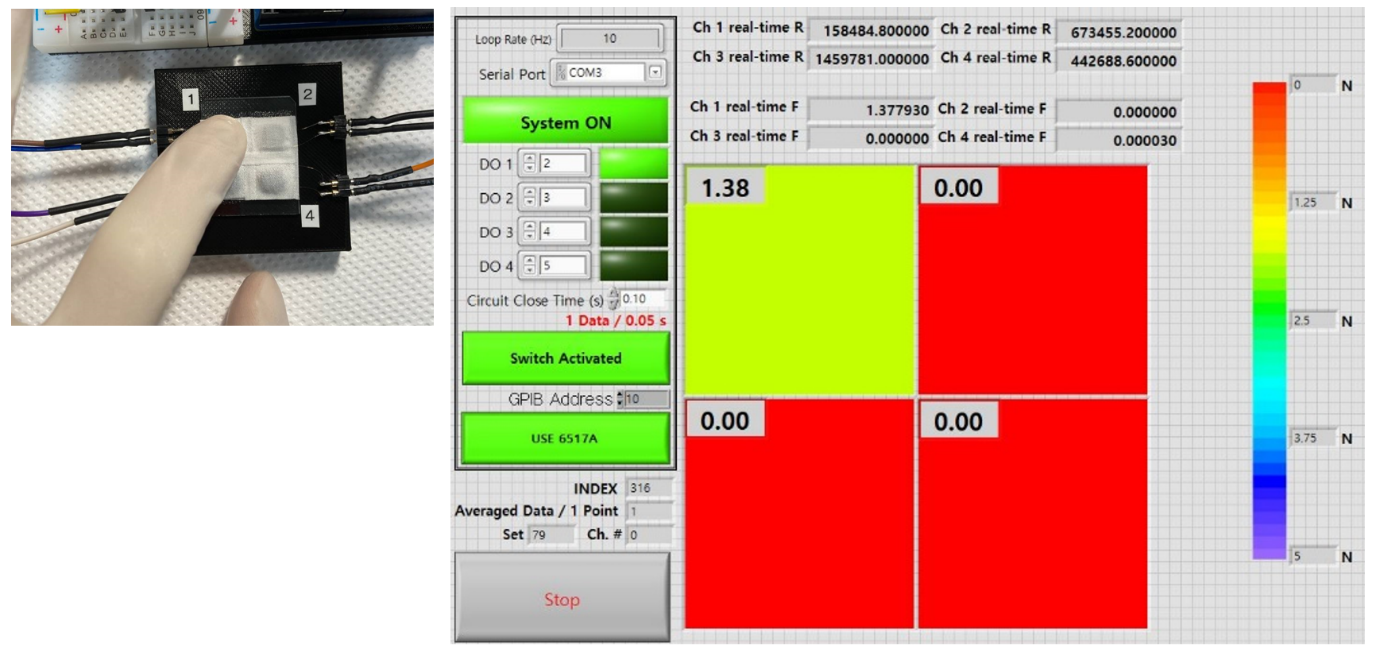


Figure S3. The operation of the pressure sensor module with Labview program.


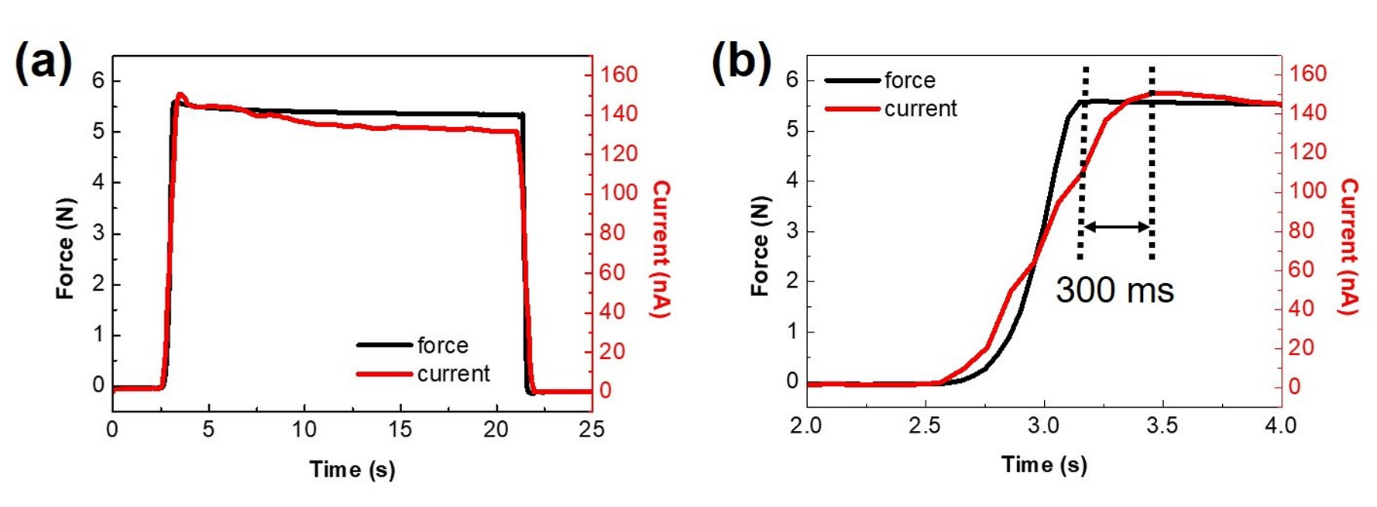


Figure S4. (a) The response time of the pressure sensor and (b) its magnified image. The response time is about 300 ms.


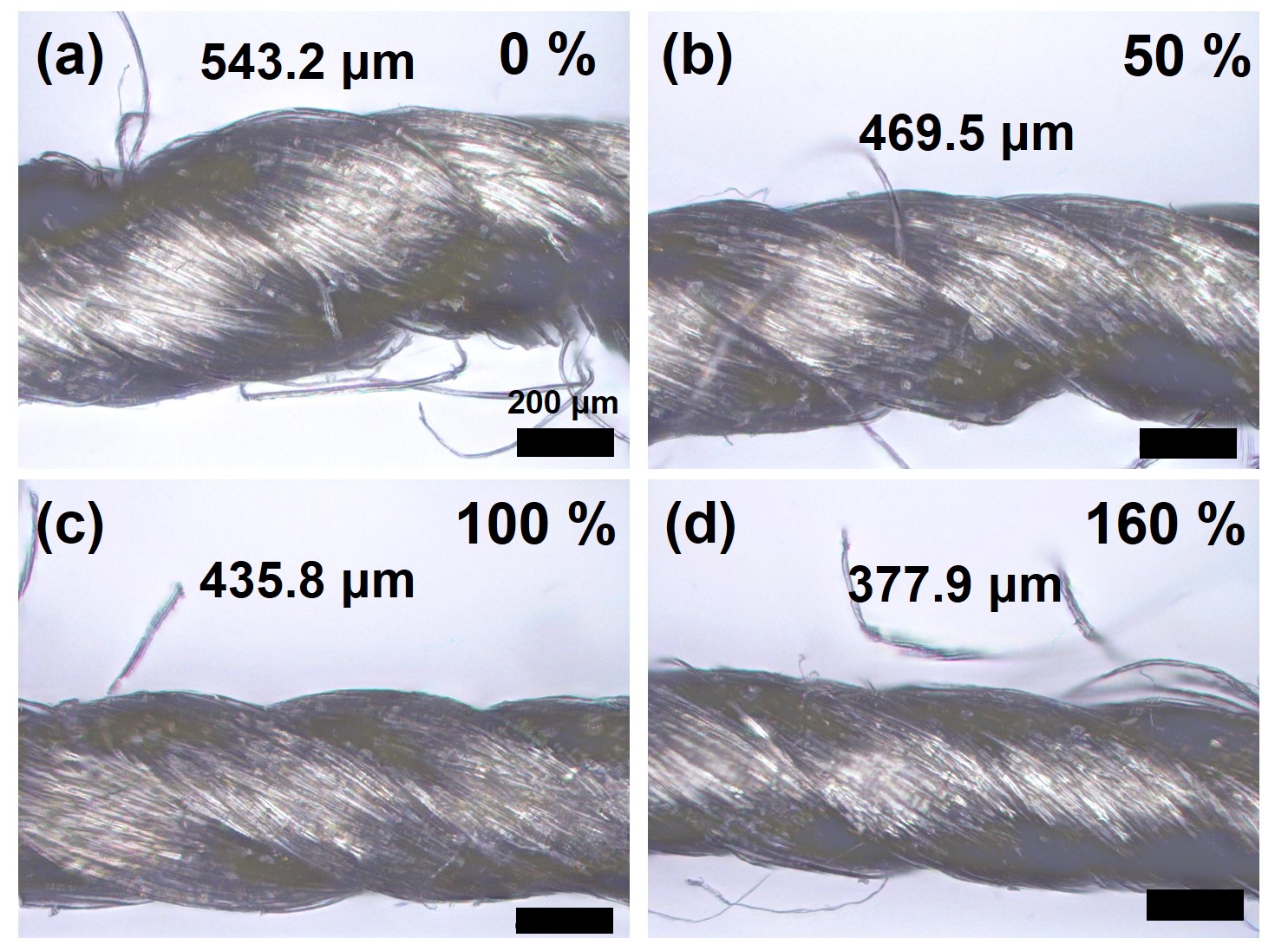


Figure S5. Strain-dependent diameter changes. All scale bars are 200 μm.


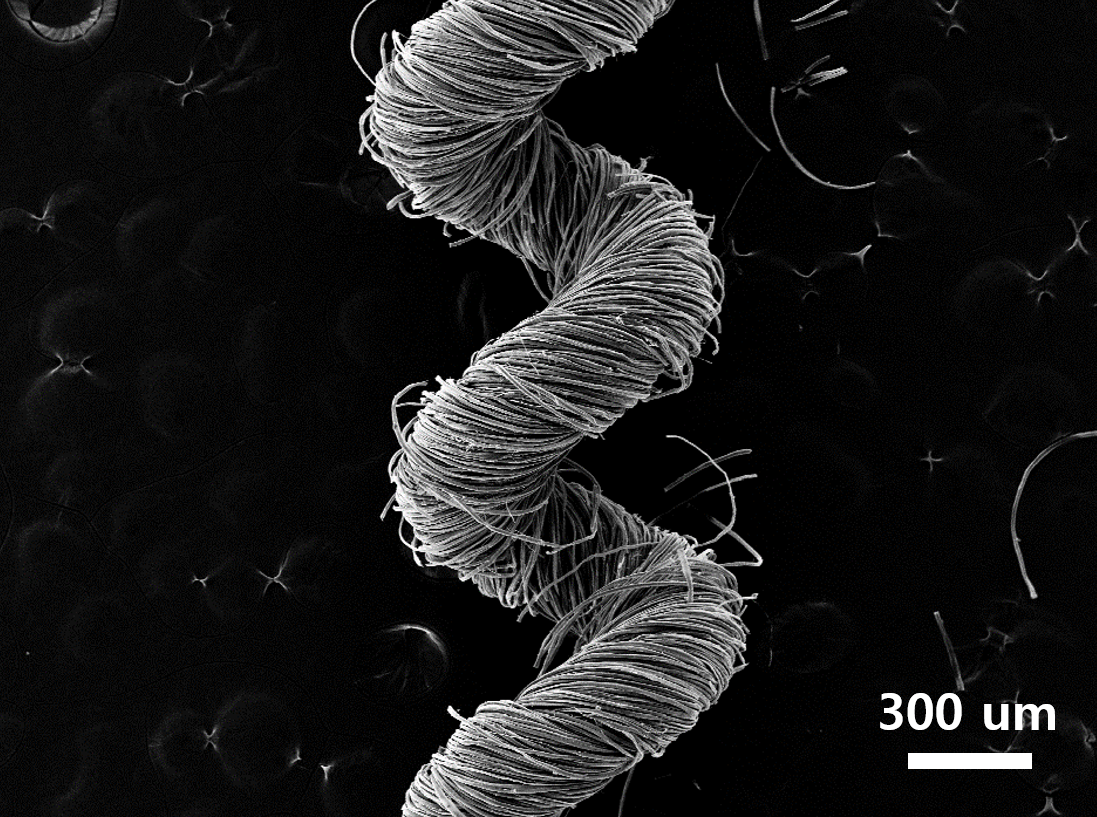


Figure S6. The SEM image of twisted rGOS yarn stretched maximally. Some fibers were broken, increasing R.


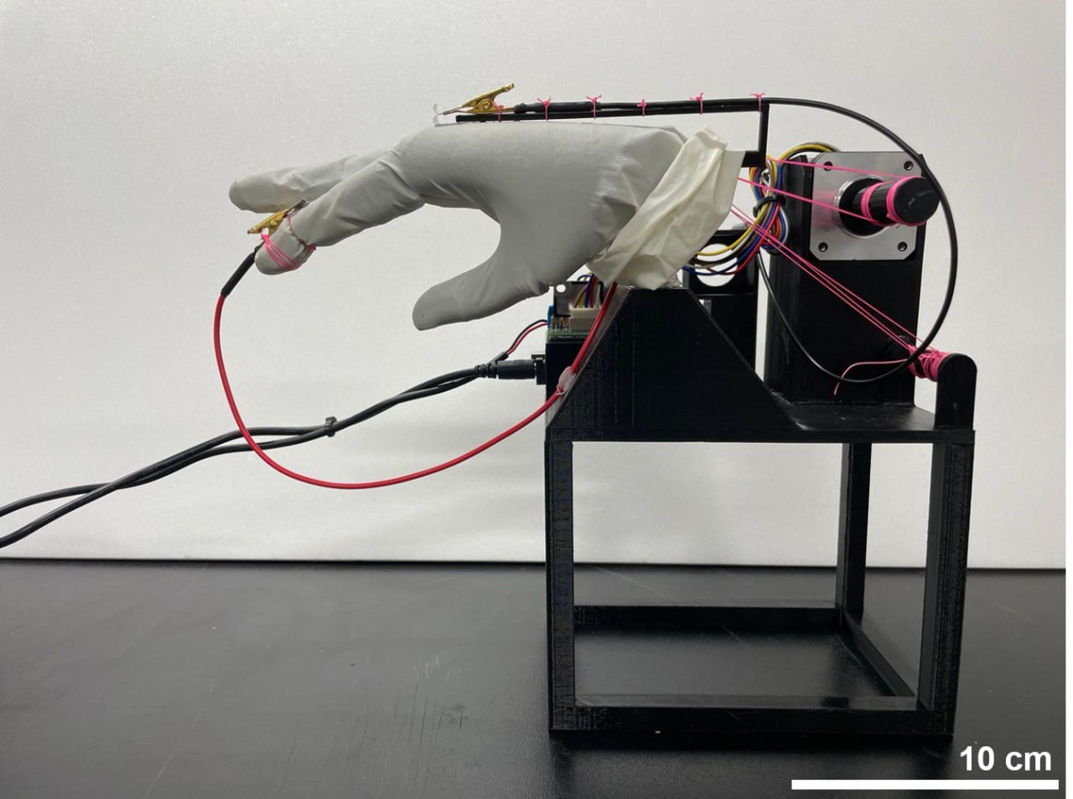


Figure S7. Home-made artificial hand using a 3D printer and step motors.
